# The Prognostic Role of Perineural Invasion for Survival in Head and Neck Squamous Cell Carcinoma: A Systematic Review and Meta-Analysis

**DOI:** 10.3390/cancers16142514

**Published:** 2024-07-11

**Authors:** Zhuo-Ying Tao, Guang Chu, Yu-Xiong Su

**Affiliations:** 1 Division of Oral and Maxillofacial Surgery, Faculty of Dentistry, The University of Hong Kong, Hong Kong SAR, China; u3006901@connect.hku.hk; 2 Division of Pediatric Dentistry and Orthodontics, Faculty of Dentistry, The University of Hong Kong, Hong Kong SAR, China; u3006909@connect.hku.hk

**Keywords:** head and neck squamous cell carcinoma, prognosis, perineural invasion, survival

## Abstract

**Simple Summary:**

Perineural invasion (PNI) is the infiltration of cancer cells into peripheral nerves and is recognized as potential cancer metastasis. The presence of PNI has been extensively studied as a prognostic factor in head and neck squamous cell carcinoma (HNSCC) recently. The aim of this study was to quantitatively assess the prognostic value of PNI in HNSCC. From the results of the systematic review and meta-analysis, we confirmed that PNI is an independent prognostic factor in HNSCC. Specifically, HNSCC patients with PNI have worse survival, and are more likely to relapse locally, regionally, and distantly compared to those without PNI. We hope that these findings can serve as a basis for consideration of PNI as an indicator for more advanced pathological stages and more intensive clinical management for HNSCC patients.

**Abstract:**

The aim of this study was to conduct a comprehensive review of the predictive significance of PNI in HNSCC survival outcomes. A systematic search was conducted across multiple databases, and all studies published in the last decade were screened (Research Registry ID: reviewregistry1853). The included studies were assessed using the Quality in Prognosis Studies tool. Survival outcome data were extracted, combined, and presented as hazard ratios (HR) with a 95% confidence interval (CI). Totally, 74 studies encompassing 27,559 patients were analyzed and revealed a cumulative occurrent rate of 30% for PNI in HNSCC. PNI+ HNSCC patients had a worse overall survival (HR: 1.91, 95% CI: 1.71–2.13), disease-specific survival (HR: 1.79, 95% CI: 1.55–2.07), disease-free survival (HR: 1.82, 95% CI: 1.69–1.96), local recurrence (HR: 2.54, 95% CI: 1.93–3.33), locoregional recurrence (HR: 2.27, 95% CI: 1.82–2.82), locoregional relapse free survival (HR: 1.77, 95% CI: 1.28–2.45), distant metastasis (HR: 1.82, 95% CI: 1.34–2.48), and distant metastasis-free survival (HR: 2.97, 95% CI: 1.82–4.85) compared to those PNI– patients. The available evidence unequivocally establishes PNI as a critical prognostic factor for worse survival in HNSCC patients.

## 1. Introduction

Head and neck squamous cell carcinoma (HNSCC) arising from the epithelial lining of the oral cavity, pharynx, hypopharynx, and larynx is the most common malignancy in the head and neck and stands as the seventh most common cancer diagnosis worldwide [1]. HNSCC accounts for around 4.5% of all malignant tumors and has a poor prognosis, with a 5-year survival rate of around 50% [2]. Various histological risk factors, including depth of invasion (DOI), positive surgical margins, high histologic grade, lymph vascular invasion (LVI), and extranodal extension (ENE), have long been identified as poor prognostic indicators in patients with HNSCC [3,4,5,6,7,8].

In addition to the above factors, perineural invasion (PNI) is also a poor prognostic risk factor for survival. PNI is the histologic detection of tumor cell infiltration into, around, or through a nerve and is a potential route of metastatic spread without enough recognition [9]. At present, the most widely accepted definition of PNI is the invasion of tumor cells within any of the three layers of the nerve sheath (endoneurium, perineurium, and epineurium) or close to a nerve and covering more than one-third of its circumference, which was proposed by Liebig et al. in 2009 [10]. PNI should not be confused with perineural spread (PNS), which is the dissemination of tumor cells along the nerve via macroscopic findings and represents symptomatic neural invasion diagnosed by magnetic resonance imaging or clinical symptoms [11]. Due to the thinness of the perineurium in small nerves, tumor cells can easily infiltrate this protective barrier. Therefore, perineural invasion (PNI) serves as a microscopic and highly sensitive indicator of neural invasion and provides additional prognostic information for the clinical management of cancer. Recent studies also used immunohistochemistry (IHC) staining of neuronal cell markers to find neural infiltration within tumor samples, such as S100, which further increased the detection rate of PNI [12,13].

Recently, more and more pathologists have consciously investigated PNI in surgically resected specimens of HNSCC and report the findings to surgeons to assist them in prognosis assessment. However, it is noteworthy that the eighth edition of the American Joint Committee on Cancer (AJCC) staging system added two histopathological features, namely DOI and ENE, to the TNM criteria, rather than PNI [14,15]. In other types of head and neck cancers, PNI affects the pathologic staging of the tumors and contributes to the decision on oncologic treatment strategy. For example, the histological detection of PNI leads to automatic T3 staging according to the staging system used for head and neck cutaneous squamous cell carcinomas [14,15]. PNI is regarded as an adverse prognostic factor for malignant salivary gland tumors and recommends a more aggressive adjuvant treatment regimen [16,17,18]. Plenty of research has reported the correlation between PNI and unfavorable clinical outcomes in HNSCC in the past decades, but still, a few studies have shown inconsistent conclusions [19,20]. Therefore, whether PNI can be used as an indicator for tumor staging or decisions on clinical management strategies in HNSCC needs compelling evidence.

Given the growing body of research on the prognostic value of PNI in HNSCC, our objective was to quantitatively assess the actual impact of PNI on survival in HNSCC patients, aiming to provide robust evidence for clinical practice and a foundation for further investigations into the mechanisms and therapeutic strategies related to PNI. The prevalence of PNI in HNSCC varies significantly (ranging from 5.2% to 90%) across different clinical studies [21]. Due to the lack of consensus on the definition and measurement methods of PNI in earlier studies, we performed a systematic review and meta-analysis of the most recent publications within the last decade to provide summary effect estimates concerning the impact of PNI on survival outcomes in HNSCC patients.

## 2. Materials and Methods

### 2.1. Search Strategy

This systematic review and meta-analysis were performed according to the Preferred Reporting Items for Systematic Reviews and Meta-Analyses (PRISMA) guideline [22] and a comprehensive literature search was conducted by ZYT on 1 January 2024 in PubMed, Web of Science, Embase, and Scopus for relevant studies using combinations of keywords from the following three domains: head and neck, squamous cell carcinoma, and perineural invasion. The detailed keywords in each domain and search strategy are shown in Table S1. All studies published between 1 January 2014 and 31 December 2023 were included for initial screening (Research Registry ID: reviewregistry1853).

### 2.2. Data Extraction and Outcome Definitions

All studies were exported from the four databases to the citation manager EndNote (version 20), and the duplicates were removed. The title and abstract of each study were screened first to exclude studies that were conference papers, reviews, or in vitro/animal studies, not in English, or without full text, or other non-relevant research. The reference lists of eligible studies were further assessed by reading the full text according to the eligibility criteria: (1) study aim/design: prospective or retrospective study of the prognostic factors including PNI in HNSCC; (2) study subjects: patients with primary HNSCC and first treated with surgery; (3) PNI classification and measurement: divided into PNI+ and PNI– subgroups and measured through the histological slides of surgically resected specimens; and (4) prognostic outcomes: reported overall survival (OS), disease-specific survival (DSS), disease-free survival (DFS), local recurrence (LR), locoregional recurrence (LRR), locoregional relapse-free survival (LRFS), distant metastasis (DM) or distant metastasis-free survival (DMFS) with hazard ratio (HR) from Cox regression models. OS was defined as the time from surgery to any-cause death, DSS to cancer-related death, DFS to cancer recurrence or death, LR to recurrence at T (the primary tumor site), LRR to recurrence at T/N (the primary tumor site and regional lymph nodes), LRFS to recurrence at T/N or death, DM to recurrence at M (metastasis at distant organs or distant lymph nodes), and DMFS to recurrence at M or death. In cases where both unadjusted and adjusted HRs were provided, the analysis utilized the adjusted values. The initial screening work and eligibility assessment were conducted by two authors (ZYT and GC) together. For included studies, the following data were extracted: the first author, year of publication, inclusion period and location of study, sample size, male proportion, age, tumor histology, TNM stages, PNI proportion, follow-up time, and prognostic outcomes in HRs with a 95% confidence interval (CI). All discrepancies in study eligibility assessment and data extraction were reviewed and determined by the corresponding author (YXS).

### 2.3. Assessment of Risk of Bias in Included Studies

The assessment of bias in the included studies was conducted using the Quality In Prognosis Studies (QUIPS) tool [23]. This tool comprises six domains: study participation, study attrition, prognostic factor measurement, outcome measurement, study confounding, and statistical analysis and reporting. Each domain consists of 3–7 specific prompts that aid in evaluating the overall risk of bias. Two authors independently assessed the risk of bias in the included studies using the QUIPS tool. The assessment of bias in each domain involved counting the number of inadequate items that were rated as “no”. Based on our predefined criteria, a domain was considered to have a “low risk” if no inadequate items were found, while a “high risk” was assigned if more than 50% of the items were inadequate. All eligible studies were included in the meta-analysis regardless of their potential risk of bias in obtaining precise results. However, the interpretation of the findings took into consideration the potential risk of bias.

### 2.4. Statistical Analysis

All analyses were performed using STATA SE 14.0 (StataCorp, College Station, TX, USA). HR and corresponding 95% CIs were used to compare the outcomes. Statistical heterogeneity among studies was calculated using the I^2^ index. The random-effects model was selected when I^2^ > 50%. Otherwise, the fixed-effects model was performed. Subgroup analysis was conducted to mitigate the potential for inconsistency. Based on the parameters of Begg’s test and Egger’s test, publication bias was assessed, and a funnel plot was created for forest plots with 10 or more studies. A *p*-value < 0.05 was considered statistically significant.

## 3. Results

### 3.1. Study Selection

**Figure 1:**
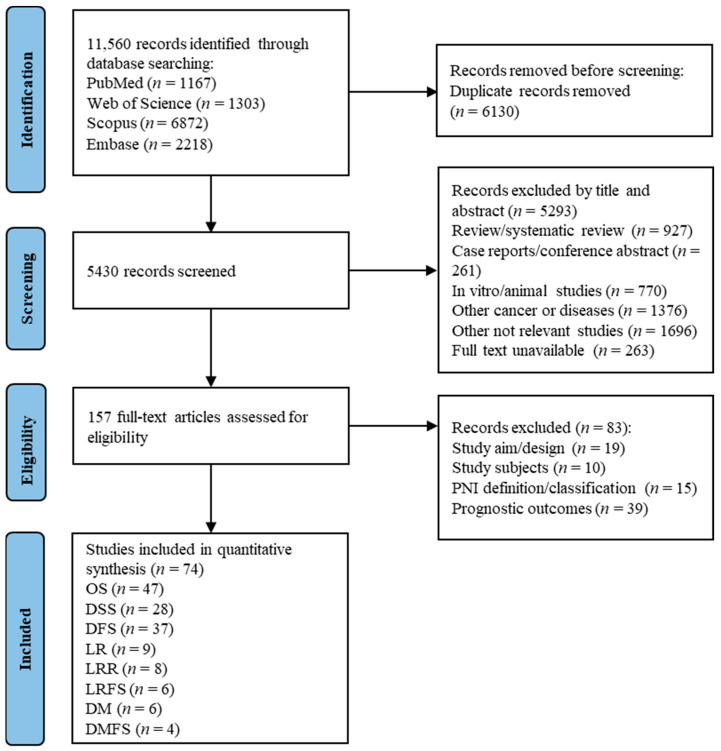
Literature search strategy and flowchart detailing study selection.

Out of the initial 11,560 records identified through database searching, 5430 records were retained after removing duplicates. During the title and abstract screening, 5293 studies were excluded from the first stage. In the second stage, the remaining 157 articles underwent a full-text review, and 83 of them were subsequently excluded due to various reasons, as shown in Figure 1. According to the predefined inclusion and exclusion criteria, 74 studies [12,19,20,24,25,26,27,28,29,30,31,32,33,34,35,36,37,38,39,40,41,42,43,44,45,46,47,48,49,50,51,52,53,54,55,56,57,58,59,60,61,62,63,64,65,66,67,68,69,70,71,72,73,74,75,76,77,78,79,80,81,82,83,84,85,86,87,88,89,90,91,92,93,94] with 77 independent cohorts were included in the present meta-analysis.

### 3.2. Characteristics of Included Studies

The characteristics of the included studies are shown in Table S2. A total of 27,559 patients included were diagnosed with HNSCC, with various primary sites including the oral cavity (89.8%), oropharynx (1.4%), hypopharynx (0.5%), and larynx (6.6%). Patient cohorts were from Asia, Europe, the Americas, and Oceania. The median number of patients per study was 215 (range 49–2082). The included study period ranged from 1985 to 2020. The median age was 57 years, with a range of 15–100 years and a male preponderance (70%). The mean follow-up time was 43.0 months, and the median follow-up time was 41 months (range 0.1–348 months). As a pooled cohort (only one cohort did not report the proportion of PNI+ patients [58], 8 045 of 26,510 patients had PNI (30%), and the median rate of PNI was 26.3% (range: 7–83.9%).

### 3.3. Risk of Bias in Included Studies

The risk of bias of all included studies was assessed using the QUIPS tool (Table S2). Only one “high risk” was rated in the study by Na’ara et al., due to the inadequate report of results, including the PNI proportion among the study subjects [58]. The other studies were rated with 1–4 “moderate risk” domains. Since all studies were retrospective cohorts, the attrition domain was not relevant, and the studies were rated “low risk”. In assessing the bias of prognostic factor measurement, only studies providing a clear definition of PNI and measurement methods can be identified as “low risk”. In assessing the bias of outcome measurement, studies should provide a clear definition of outcome variables. Any study reporting without the follow-up time or measurement methods of outcome variables was rated as “moderate risk”. In order to mitigate the impact of confounding factors in the study, it is important to consider and address potential confounders in the analysis. This can be achieved by employing techniques such as utilizing the multivariable Cox regression model to adjust for the prognostic significance of PNI. For data analysis and reporting, an appropriate statistical model should be applied, and enough primary and secondary results should be presented to support the conclusion.

### 3.4. Prognostic Role of PNI for Survival in HNSCC

#### 3.4.1. OS

**Figure 2:**
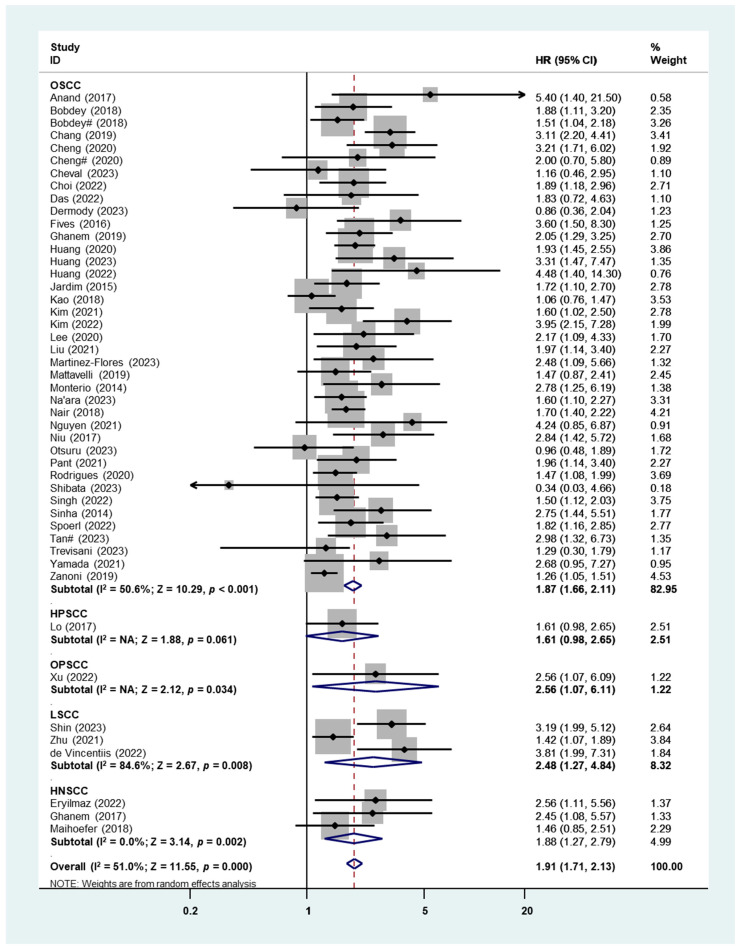
Meta-analysis of the prognostic role of PNI for OS in HNSCC using the random-effects analysis (# distinguishes the study with same first author name and publication year).

Forty-seven studies comprising 47 patient cohorts were examined to assess the prognostic significance of PNI for OS in patients with HNSCC (Figure 2). Due to the significant heterogeneity (I^2^ = 51.0%), a random-effects analysis was performed. As shown in Figure 2, PNI+ patients had significantly worse OS compared to PNI– patients (HR: 1.91, 95% CI: 1.71–2.13, *p* < 0.001). Subgroup analyses were performed based on various primary cancer sites, revealing that the prognostic significance of PNI for overall survival OS was significant, specifically in cases of oral squamous cell carcinoma. (OSCC) (HR: 1.87, 95% CI: 1.66–2.11, *p* < 0.001) and laryngeal squamous cell carcinoma (LSCC) (HR: 2.48, 95% CI: 1.27–4.84, *p* = 0.008). Only one study focused on oropharyngeal squamous cell carcinoma (OPSCC), in which the prognostic role of PNI was significant (HR: 2.56, 95% CI: 1.07–6.11, *p* = 0.034), while the other study focused on hypopharyngeal squamous cell carcinoma (HPSCC), in which the prognostic role of PNI was not significant (HR: 1.61, 95% CI: 0.98–2.65, *p* = 0.061) (Figure 2). Meanwhile, significant findings were also detected in the subgroup analyses.

Subgroup analysis in locations showed that the worst OS was found in Europe (HR = 2.01, 95% CI: 1.47–2.76, *p* < 0.001), followed by Asia (HR = 1.98, 95% CI: 1.71–2.30, *p* < 0.001), Oceania (HR = 1.97, 95% CI: 1.14–3.40, *p* = 0.015), and Americas (HR = 1.70, 95% CI: 1.34–2.16, *p* < 0.001). Interestingly, the presence of PNI was associated with worse OS in HNSCC patients with advanced stages (HR = 2.04, 95% CI: 1.50–2.79, *p* < 0.001), but not in those with early stages (HR: 1.97, 95% CI: 0.90–4.32, *p* = 0.091) (Table 1). Publication bias was assessed using Begg’s funnel plot and Egger’s test, which revealed the presence of significant publication bias (Begg’s test: *p* = 0.015; Egger’s test: *p* = 0.012) (Supplementary Figure S1). When conducting a meta-analysis that includes only studies with positive or favorable results, the funnel plot depicting the individual studies may exhibit asymmetry. This asymmetry is anticipated in the case of PNI due to the small number of published studies that have null findings when comparing outcomes between patients with and without PNI. This is primarily attributed to the significant impact of PNI on patient survival, making it less likely to find studies with neutral or negative conclusions.

#### 3.4.2. DSS

**Figure 3:**
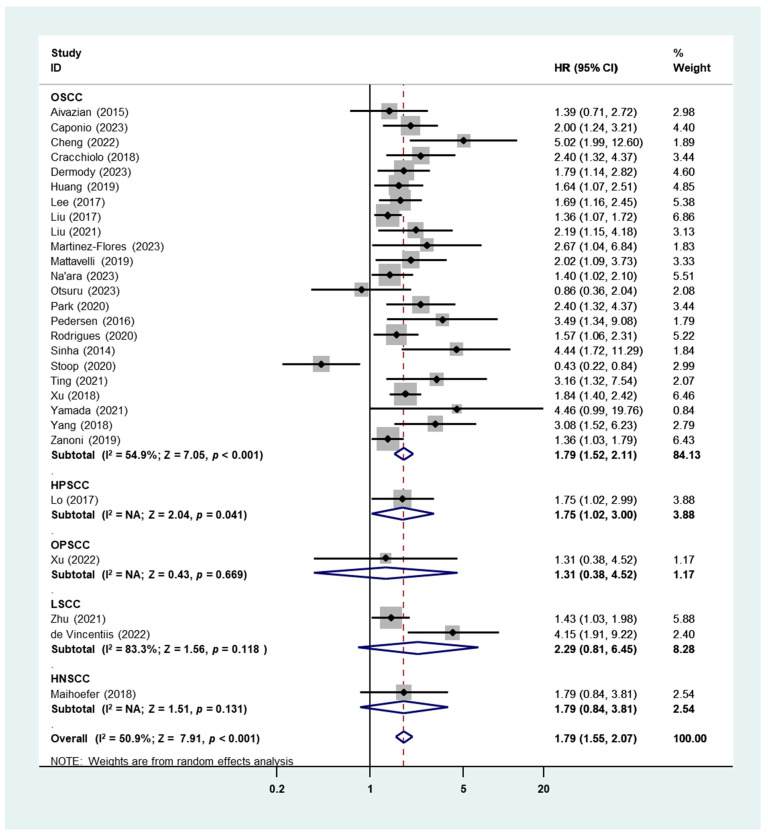
Meta-analysis of the prognostic role of PNI for DSS in HNSCC using the random-effects analysis.

A total of 28 studies involving 28 cohorts, primarily comprising patients with oral squamous cell carcinoma (OSCC), were pooled together for the analysis of the prognostic significance of perineural invasion (PNI) for disease-specific survival (DSS) in head and neck squamous cell carcinoma (HNSCC) (Figure 3). Given the considerable heterogeneity observed (I^2^ = 50.9%), a random-effects model was utilized. Based on the combined results presented in Figure 3, it was found that patients with PNI exhibited a poorer DSS compared to those without PNI. (HR: 1.79, 95% CI: 1.55–2.07, *p* < 0.001). Only five studies were not focused on OSCC, namely, one on HPSCC (HR: 1.75, 95% CI: 1.02–3.00, *p* = 0.041), one on OPSCC (HR: 1.31, 95% CI: 0.38–4.52, *p* = 0.669), two on LSCC (HR: 2.29, 95% CI: 0.81–6.45, *p* = 0.118), and one on HNSCC (HR: 1.79, 95% CI: 0.84–3.81, *p* = 0.131) (Figure 3).

Subgroup analysis in locations showed that the worst DSS was found in Oceania (HR = 1.98, 95% CI: 1.37–2.86, *p* < 0.001), followed by the Americas (HR = 1.78, 95% CI: 1.38–2.29, *p* < 0.001) and Asia (HR = 1.76, 95% CI: 1.45–2.14, *p* = 0.015), while Europe studies exhibited a marginal effect of PNI in DSS (HR = 1.84, 95% CI: 1.00–3.39, *p* = 0.051) (Table 1). Unlike OS, the presence of PNI was associated with worse DSS in HNSCC patients with both early stages (HR: 2.59, 95% CI: 1.25–5.40, *p* = 0.011) and advanced stages (HR = 2.64, 95% CI: 1.52–4.59, *p* = 0.001) (Table 1). Begg’s funnel plot and Egger’s test indicated a significant publication bias (Begg’s test: *p* = 0.002; Egger’s test: *p* = 0.011) (Figure S1B).

#### 3.4.3. DFS

**Figure 4:**
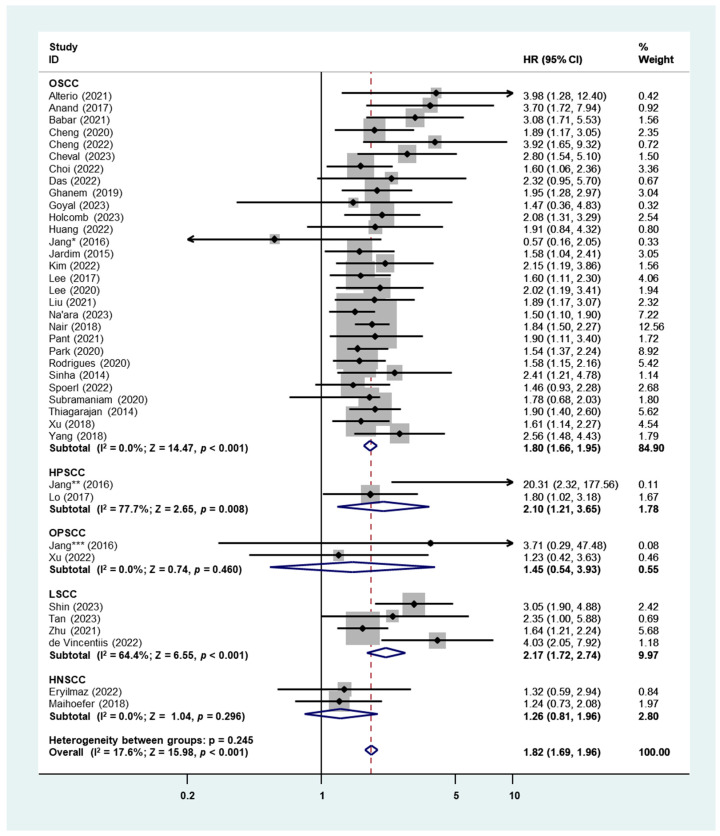
Meta-analysis of the prognostic role of PNI for DFS in HNSCC using the fixed-effects analysis. Jang * (2016), Jang ** (2016) and Jang *** (2016) are three cohorts from the same study Jang et al. [92].

There are 37 studies with 39 patient cohorts in the investigation of the prognostic role of PNI for DFS in HNSCC. The fixed-effects model was used due to the low heterogeneity (I^2^ = 17.6%). As the pooled result displayed in Figure 4 shows, PNI+ patients had a significantly more unfavorable DFS than PNI- patients (HR: 1.82, 95% CI: 1.69–1.96, *p* < 0.001). Subgroup analysis in histology indicated that the worst DFS was found in LSCC (HR: 2.17, 95% CI: 1.72–2.74, *p* < 0.001), followed by HPSCC (HR: 2.10, 95% CI: 1.21–3.65, *p* = 0.008) and OSCC (HR: 1.80, 95% CI: 1.66–1.95, *p* < 0.001), while the presence of PNI showed no significant prognostic role of DFS in OPSCC (HR: 1.45, 95% CI: 0.54–3.93, *p* = 0.460) (Figure 4).

Similar to DSS, PNI leading to worse DFS was found in all locations (Asia, HR: 1.88, 95% CI: 1.70–2.08, *p* < 0.001; Americas, HR: 1.83, 95% CI: 1.51–2.23, *p* < 0.001; Europe, HR: 1.96, 95% CI: 1.50–2.55, *p* < 0.001; Oceania, HR: 1.61, 95% CI: 1.29–2.00, *p* < 0.001) and stages (early stages, HR: 2.44, 95% CI: 1.85–3.22, *p* < 0.001; advanced stages, HR: 1.95, 95% CI: 1.51–2.52, *p* < 0.001; all stages, HR: 1.77, 95% CI: 1.63–1.91, *p* < 0.001) (Table 1). Begg’s funnel plot and Egger’s test were employed to identify publication bias, and a significant publication bias was detected (Begg’s test: *p* = 0.009; Egger’s test: *p* = 0.006) (Figure S1C).

#### 3.4.4. LR/LRR/LRFS

**Figure 5:**
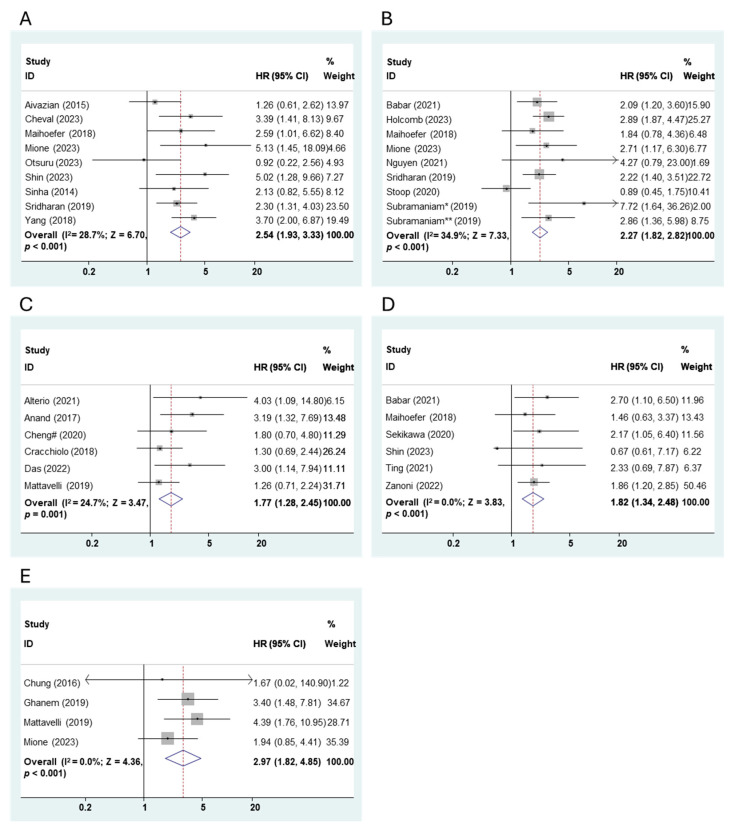
Meta-analysis of the prognostic role of PNI for (**A**) LR, (**B**) LRR, (**C**) LRFS, (**D**) DM and (**E**) DMFS in HNSCC using the fixed-effects analysis (# distinguishes the study with same first author name and publication year; Subramaniam* (2019) and Subramaniam** (2019) are two cohorts from the same study Subramaniam et al. [73]).

There are 9 studies with 9 cohorts, 8 studies with 9 cohorts, and 6 studies with 6 cohorts in the investigation of the prognostic role of PNI for LR, LRR, and LRFS in HNSCC. Fixed-effects models were applied due to the low heterogeneity (LR: I^2^ = 24.7%; LRR: I^2^ = 0.0%; LRFS: I^2^ = 0.0%). According to the pooled results in Figure 5A,B, PNI was associated with a higher risk of both LR (HR: 2.54, 95% CI: 1.93–3.33, *p* < 0.001) and LRR (HR: 2.27, 95% CI: 1.82–2.82, *p* < 0.001) in HNSCC. Obviously, PNI+ patients held a worse LRFS when compared to PNI– patients (HR: 1.77, 95% CI: 1.28–2.45, *p =* 0.001) (Figure 5C).

#### 3.4.5. DM/DMFS

A total of 6 studies with 6 cohorts and 4 studies with 4 cohorts were included in the analysis of the prognostic role of PNI for DM and DMFS in HNSCC. Fixed-effects models were applied due to the low heterogeneity (DM: I^2^ = 0.0%; DMFS: I^2^ = 0.0%). As indicated in the pooled results in Figure 5D,E, PNI increased the risk of DM (HR: 1.82, 95% CI: 1.34–2.48, *p <* 0.001) and thus led to worse DMFS (HR: 2.97, 95% CI: 1.82–4.85, *p <* 0.001) in HNSCC.

## 4. Discussion

PNI has a frequent occurrence in HNSCC, but the prevalent rate of PNI varies largely between different studies. According to our meta-analysis, the PNI detection rate ranges from 7% to 83.9%, with an average rate of around 30%. Notably, advanced tumors have higher rates of PNI than those early-stage ones. Specifically, Bobdey et al. reported the highest rate of PNI (83.9%) among all included studies, which may be attributed to the included subjects being OSCC patients with T4 stage [28]. Cheng et al. presented the lowest PNI rate (7%) as the study focused on early tongue cancer [34]. Potential selection bias could also be a reason because of the retrospective design of the included studies. Moreover, the assessment for PNI by reviewing pathology slides or records influences the PNI prevalence. Due to the updated criteria, previous studies may underestimate PNI, depending on when the histological slides were assessed. Generally, most pathologists estimated the presence of PNI through histological slides with H&E staining, but some small nerve fibers were difficult to distinguish from tumor tissues, and it may challenge pathologists without a lot of experience [13]. Some recent studies have introduced IHC staining of neuronal cell markers to decrease the difficulty of measurement and increase the detection rate of PNI [12,95]. In this study, all included publications provided specific survival outcomes for PNI based on a histologic definition, with the exception of Martinez-Flores et al. [12], which detected PNI by immunochemistry staining of S100. A sensitivity analysis was conducted, considering both the inclusion and exclusion of data from this study, which did not affect conclusions, and thus the publication was included in the final analysis. However, a systematic review comparing IHC staining and H&E staining in the detection of PNI in OSCC reported that the sensitivity and specificity of IHC staining using neural biomarkers were 76% and 42%, respectively, and the overall accuracy was found to be only 58% due to high inter-study heterogeneity [13]. These findings highlight the need for additional research to validate the role of IHC staining in PNI detection. In the future, PNI will be considered a harbinger of tumor dissemination and metastasis owing to the more precise and efficient detection of PNI at an early stage of cancer development.

In the present study, we demonstrated that there was a significant pooled HR for OS, DSS, and DFS in patients with HNSCC with PNI compared to those without, and the subgroup analysis results indicated that the location, tumor stage, sample size, and PNI% did not affect the above results (Table 1). The heterogeneities of all analyses fell within the accepted limits, with the highest I^2^ equal to 51.0% (OS). However, in the histology subgroup analysis, the pooled HR of OS in HPSCC (Figure 2), DSS in OPSCC, LSCC, and HNSCC (Figure 3), as well as DFS in OPSCC and HNSCC (Figure 4), were not significant. This was due to the extremely small number of studies included in these subgroups—only one or two studies. Therefore, the overall HR of these survival outcomes was ineluctably affected by studies focusing on OSCC patients, which shared more than 80% weight in each analysis. Only two studies presented the prognostic outcomes of OPSCC and indicated a significant HR of OS but not in DSS or DFS [84,92]. Basically, the infection status of HPV significantly affects the prognosis of OPSCC [96], but the above studies fail to add HPV status as an adjusted factor in multiple Cox regression analyses for survival, which may lead to a bias in the results. Only two studies reported the survival data of HPSCC, which demonstrated significant HRs of DSS and DFS and a critical HR of OS [86,92]. Similarly, in LSCC, all four included studies indicated worse survival in PNI+ patients [85,87,88,89], though the pooled HR for DSS was not significant due to the high heterogeneity (Figure 3) [85,89]. Our findings bring additional value and a robust base for the fact that PNI is an independent adverse prognostic indicator for survival outcomes in HNSCC, and we hope that there will be more work in HNSCC other than OSCC in order to enrich the results and consolidate our conclusion.

Our results also indicated that the pooled HR for LR, LRR, LRFS, DM, and DMFS in HNSCC patients was significant (Figure 5), which provided evidence that PNI is a risk factor for local, locoregional, and distant relapse. Actually, the potential value of PNI as a route of tumor cell spreading is underrecognized in HNSCC clinically. PNI usually starts with small nerve branches within the tumor or in peritumoral spaces and then progresses to larger ones. When tumor cells disseminate along the nerves, some tumor cells can “skip” to a far point from the primary site, sometimes even exceeding the normal surgical margin [11]. This phenomenon may contribute to the local recurrence of tumors. Regarding the pathological evidence linking PNI and regional or distant metastasis, there is a need for additional research to fill this research gap.

Recently, several systematic reviews have highlighted the significant prognostic role of PNI in HNSCC. For instance, Binmadi et al. [97] focused on OSCC, while Li et al. [98] focused specifically on tongue squamous cell carcinomas. These studies, along with our own, have collectively validated the predictive value of PNI in survival by synthesizing the HRs from the Cox regression models of the included studies. It is worth noting that Binmadi et al. and Li et al. specifically examined the subtype of HNSCC, whereas our study encompassed all HNSCC studies. Although studies on non-oral site HNSCC remain limited, our research aims to raise awareness regarding the importance of considering PNI in these specific HNSCC subtypes, benefiting both clinicians and researchers. Furthermore, our study includes the most recent research up to 2023, providing an updated perspective. In contrast, the aforementioned studies only included studies up to 2020, thus highlighting the timeliness and relevance of our findings. In addition to investigating the role of PNI in survival, we also focused on the predictive value of PNI in local, regional, and distant recurrences of HNSCC. Our findings provide compelling evidence supporting PNI as a potential mechanism for tumor dissemination. By addressing these aspects comprehensively, our study contributes to the current understanding of PNI in HNSCC, emphasizing its prognostic significance and potential implications for disease management.

Furthermore, the predictive significance of PNI may be partially reliant on the assessment and classification methods employed. Some studies also set subgroups of patients by subcategorization of PNI, such as the number of foci and the location of neural invasion. Tumors with multifocal PNI have worse survival than those without PNI [30,37,99,100,101], while the prognostic role of unifocal PNI in HNSCC remains controversial since three studies hold a significant conclusion [30,37,99] but the other two reported HRs of survival outcomes with no significance [100,101]. Some studies also set the cut-off value of PNI foci at 4 or 5, which leads to the conclusion that more foci of PNI within the tumor sample lead to worse survival [102,103]. This is consistent with another study indicating that intratumoral nerve density is associated with adverse clinical outcomes in tumors [104]. As for the location of PNI, it seems that peritumoral or extratumoral PNI has a more adverse effect on the prognosis of HNSCC than intratumoral invasion of nerves [12,63,101,105,106]. Other factors, such as PNI involving multiple nerves [24,63] or larger-diameter nerves [95], resulted in worse survival. Nevertheless, it is essential to conduct further research to validate these findings.

Recently, there has been a more in-depth exploration of the underlying mechanisms that contribute to the interaction between nerves and cancer, as well as how nerve–cancer crosstalk promotes tumor aggressiveness. In the tumor microenvironment, neuronal cells and tumor cells interact directly, and the denervation of tumors leads to a decrease in tumor growth [107,108]. Many different neuroactive molecules and cellular processes, including neurotransmitters, neurotrophic factors, neuropeptides, axonal guidance factors, and neurogenesis, are involved with this process. A recent study reported that sensory nerves accelerated tumor growth by releasing calcitonin gene-related peptides, which affected the adaptive immune system within the tumor microenvironment [109]. Considering the aforementioned information, it becomes apparent that the assessment of PNI should be regarded not only as a prognostic factor for the survival of HNSCC but also as a promising area for future research on innovative adjuvant therapeutic agents targeting nerve-cancer crosstalk.

We have to acknowledge that there are some limitations in our present study. Firstly, our meta-analysis includes pooled data from all histologic types of HNSCC, but a majority of the studies focused on OSCC. It is our aspiration that this study will serve as a catalyst for future investigations into the prognostic significance of PNI in OPSCC, HPSCC, and LSCC. By expanding the sample size within each category, subsequent analyses could potentially yield more comprehensive survival data, leading to a more precise and conclusive understanding of the prognostic value of PNI in HNSCC. Secondly, this meta-analysis only included nonrandomized, retrospective studies, so publication bias is inherent according to the funnel plots. One potential explanation could be the scarcity of studies reporting null findings in the literature, which is not surprising considering the recognized aggressiveness and lethality associated with PNI. Thirdly, our meta-analysis includes studies conducted in various countries, and some studies had a long time span of patient inclusion. Slight variations in PNI definition and detection were inevitable. This can be attributed to the utilization of different guidelines by the participating institutions and pathologists. Also, advancements and improvements in treatment modalities have occurred over the years when comparing the 1980s to the 2010s, which may potentially influence survival outcomes.

## 5. Conclusions

In conclusion, our meta-analysis provides compelling evidence supporting the poor survival implications of PNI in HNSCC. As the most recent source of evidence, the results of our study can serve as a valuable resource for facilitating discussions among patients, oral and maxillofacial surgeons, otolaryngologists, radiation therapists, and oncologists regarding prognosis and long-term care. We hope that our findings can serve as a basis for potential revisions to the upcoming edition of the AJCC staging criteria, provided there is substantial justification supported by additional studies, with the ultimate goal of enhancing patient care. In the future, there is a possibility that PNI, together with other histological factors, may be considered as pathological staging criteria, leading to the assignment of higher tumor stages for cancer exhibiting these high-risk histological factors.

## Figures and Tables

**Table 1:** Subgroup analysis of OS, DSS, and DFS.

Domain	Subgroup	OS				DSS				DFS			
		No. Studies	HR (95%)	*p*-Value	Hetero-Geneity	No. Studies	HR (95%)	*p*-Value	Hetero-Geneity	No. studies	HR (95%)	*p*-Value	Hetero-Geneity
Location	Asia	27	1.98 (1.71, 2.30)	***	I^2^ = 54.5%	11	1.76 (1.45, 2.14)	***	I^2^ = 46.1%	24	1.88 (1.70, 2.08)	***	I^2^ = 3.9%
	Americas	10	1.70 (1.34, 2.16)	***	I^2^ = 46.1%	7	1.78 (1.38, 2.29)	***	I^2^ = 33.0%	6	1.83 (1.51, 2.23)	***	I^2^ = 13.2%
	Europe	7	2.01 (1.47, 2.76)	***	I^2^ = 42.1%	6	1.84 (1.00, 3.39)	0.051	I^2^ = 79.2%	5	1.96 (1.50, 2.55)	***	I^2^ = 66.2%
	Oceania	1	1.97 (1.14, 3.40)	0.015	I^2^ = NA	3	1.98 (1.37, 2.86)	***	I^2^ = 0.0%	2	1.61 (1.29, 2.00)	***	I^2^ = 0.0%
Stage	Early stages	5	1.97 (0.90, 4.32)	0.091	I^2^ = 59.6%	4	2.59 (1.25, 5.40)	0.011	I^2^ = 65.7%	6	2.44 (1.85, 3.22)	***	I^2^ = 0.0%
	Advanced stages	6	2.04 (1.50, 2.79)	***	I^2^ = 49.5%	3	2.64 (1.52, 4.59)	0.001	I^2^ = 43.7%	4	1.95 (1.51, 2.52)	***	I^2^ = 45.3%
	All stages	36	1.90 (1.68, 2.13)	***	I^2^ = 52.4%	21	1.64 (1.43, 1.88)	***	I^2^ = 40.9%	30	1.77 (1.63, 1.91)	***	I^2^ = 14.6%
Sample size	≥500	8	1.39 (1.23, 1.57)	***	I^2^ = 19.0%	6	1.81 (1.50, 2.19)	***	I^2^ = 0.0%	6	1.74 (1.54, 1.95)	***	I^2^ = 0.0%
	<500	39	2.12 (1.90, 2.38)	***	I^2^ = 27.5%	22	1.81 (1.51, 2.17)	***	I^2^ = 58.7%	33	1.87 (1.71, 2.01)	***	I^2^ = 24.6%
PNI%	≥30	20	1.63 (1.41, 1.89)	***	I^2^ = 38.1%	10	1.68 (1.28, 2.19)	***	I^2^ = 61.0%	11	1.80 (1.56, 2.08)	***	I^2^ = 0.0%
	<30	26	2.17 (1.87, 2.53)	***	I^2^ = 49.4%	17	1.92 (1.59, 2.31)	***	I^2^ = 47.3%	27	1.87 (1.71, 2.04)	***	I^2^ = 26.9%

Key: OS: overall survival. DSS: disease-specific survival. DFS: disease-free survival. HR: hazard ratio. PNI: perineural invasion. ***: *p* < 0.001.

## Data Availability

All data relevant to this study are contained within the manuscript. No additional data beyond those presented in the manuscript can be provided.

## Supplementary Materials

The following supporting information can be downloaded at: https://www.mdpi.com/article/10.3390/cancers16142514/s1, Table S1: Searching Strategies; Table S2: Overview of Included Studies; Figure S1: Funnel plot showing publication bias for included studies in meta-analysis for OS, DSS and DFS.
