# Supplementary material for: The Prognostic Role of Perineural Invasion for Survival in Head and Neck Squamous Cell Carcinoma: A Systematic Review and Meta-Analysis
Source: Cancers (Basel). 2024 Jul 11;16(14):2514. doi: 10.3390/cancers16142514 (PMC11274576; doi:10.3390/cancers16142514)
Supplement: Supplementary file 1 [file cancers-16-02514-s001.zip › Figure S1.pdf]

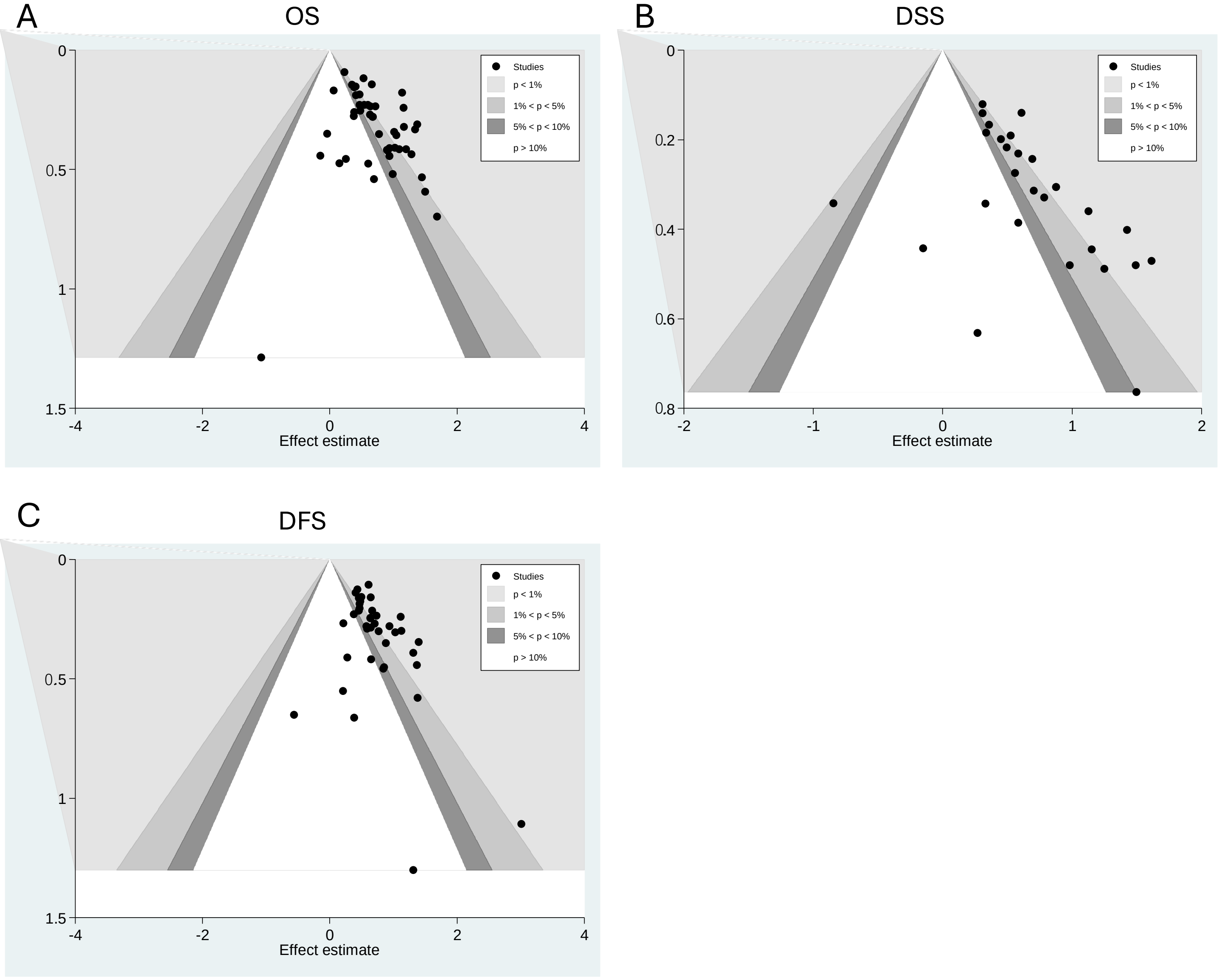

Supplementary Figure S1. Funnel plot showing publication bias for included studies in meta-analysis for (A) OS, (B) DSS and (C) DFS.
