# Supplementary material for: The Prognostic Role of Perineural Invasion for Survival in Head and Neck Squamous Cell Carcinoma: A Systematic Review and Meta-Analysis
Source: Cancers (Basel). 2024 Jul 11;16(14):2514. doi: 10.3390/cancers16142514 (PMC11274576; doi:10.3390/cancers16142514)
Supplement: Supplementary file 1 [file cancers-16-02514-s001.zip › Table S1.pdf]

Supplementary Table S1. Searching strategies.

Domains		Keywords
Head and neck	#1	head and neck
	#2	lip
	#3	oral
	#4	mouth
	#5	tongue
	#6	buccal mucosa
	#7	alveolar ridge
	#8	retromolar trigone
	#9	gingiv*
	#10	palate
	#11	pharyn*
	#12	oropharyn*
	#13	tonsil
	#14	posterior pharyngeal wall
	#15	hypopharyn*
	#16	laryn*
	#17	ethmoid sinus
	#18	maxillary sinus
Squamous cell carcinoma	#19	Squamous cell carcinoma
	#20	cancer
	#21	malignan*
	#22	neoplasm*
	#23	tumor
	#24	tumour
	#25	carcinoma*
Perineural invasion	#26	perineural invasion

Searching strategy: (1 OR 2 OR 3 OR 4 OR 5 OR 6 OR 7 OR 8 OR 9 OR 10 OR 11 OR 12 OR 13 OR 14 OR 15 OR 16 OR 17 OR 18) AND (19 OR 20 OR 21 OR 22 OR 23 OR 24 OR 25) AND 26
Key: gingiv* means all words containing "gingiv" including gingiva, gingival, etc, and so as other keywords with *.
