# Supplementary material for: The Prognostic Role of Perineural Invasion for Survival in Head and Neck Squamous Cell Carcinoma: A Systematic Review and Meta-Analysis
Source: Cancers (Basel). 2024 Jul 11;16(14):2514. doi: 10.3390/cancers16142514 (PMC11274576; doi:10.3390/cancers16142514)
Supplement: Supplementary file 1 [file cancers-16-02514-s001.zip › Table S2.pdf]

Supplementary Table S2: Overview of Included Studies.

Author	Year	Location	Inclusion Period	Sample size	Male %	Mean age, y (range/ SD)	Histo-logy	Stage (guide-line)	Adjuvant treatment	Mean follow up, m (range/ SD)	PNI %	Effect size	QUIP					
													A	B	C	D	E	F
Aivazian	2015	Australia	1995-2010	318	61	64* (30-92)	OSCC	All AJCC7	RT, CT	NR	12.5	LR, DSS	M	L	L	L	L	L
Alterio	2021	Italy	2014-2019	94	51	63* (43-73)	OSCC	pT1-2, c/pN0 AJCC7	RT, CT	24* (0.5-68)	16	DFS, LRFS	L	L	M	L	L	L
Anand	2017	India	2009-2012	83	84	21-80	OSCC	All AJCC8	RT, CRT	31* (2.8-72)	21.6	DFS, LRFS, OS	L	L	M	L	L	L
Babar	2021	USA	2005-2015	196	63.3	56*	OSCC	All AJCC7	CRT	NR	65.6	DFS, DM	M	L	M	L	M	L
Bobdey	2018	India	2009-2014	205	82.4	50* (26-73)	OSCC	T4 AJCC7	NR	21* (2-68)	83.9	OS	L	L	M	L	L	L
Bobdey#	2018	India	2006-2008	409	70.9	52* (24-85)	OSCC	All NR	RT, CT	41* (1-103)	27.8	OS	M	L	M	L	L	L
Caponio	2023	Italy	1999-2013	200	63	64.6 (14.0)	OSCC	All AJCC8	RT, CT	51.81 (36.40)	81	DSS	L	L	L	L	L	L
Chang	2019	Taiwan	2002-2015	341	91.8	52.1 (23-84)	OSCC	All AJCC8	RT, CT	50.5 (35.7)	19.9	OS	L	L	M	L	M	L
Cheng	2020	Taiwan	2002-2011	191	92.7	50.1 (10.4)	OSCC	IV AJCC7	RT, CT	29.8* (1.5-132.0)	56.1	OS, DFS	L	L	M	M	L	L
Cheng#	2020	Taiwan	2007-2014	199	82.9	52* (27-86)	OSCC	T1-2N0 NR	N	89*	7	OS, LRFS	M	L	M	M	L	L
Cheng	2022	Taiwan	2012-2017	166	89.2	56* (33-92)	OSCC	T2N0 AJCC7	RT, CT	43.5* (2.3-101.6)	28	DSS, DFS	L	L	M	L	L	L
Cheval	2023	France	2006-2021	123	50.4	75* (20-93)	OSCC	All AJCC8	RT, CT	45*	30.8	OS, DFS, LR	L	L	M	L	M	L
Choi	2022	Korea	2001-2020	407	64.1	NR	OSCC	All AJCC8	RT, CT	NR	16.1	OS, DFS	M	L	L	L	L	L
Chung	2016	Korea	1995-2014	85	95.3	58* (18-78)	OPSCC	All AJCC7	RT, CRT	48* (5.3-189.2)	7.1	DMFS	L	L	M	L	L	M

Cracchiolo	2018	USA	2000-2012	381	58.3	57* (18-96)	OSCC	All NR	RT, CT	39.8* (0.03-150.1)	28	DSS, LRFS	L	L	M	L	L	L
Das	2022	India	2011-2017	104	77.9	52.5* (27-81)	OSCC	cT4b AJCC7	RT, CT	28* (0.5-76.5)	54.8	LRFS, DFS, OS	L	L	M	L	L	L
de Vincentiis	2022	Italy	2005-2018	170	93.5	62.5 (33-79)	LSCC	T2-4a AJCC8	NR	NR	26.4	OS, DSS, DFS	M	L	M	M	L	M
Dermody	2023	USA	1998-2017	342	59	59*	OSCC	All AJCC8	NR	NR	43.9	DSS, OS	M	L	M	M	L	L
Eryilmaz	2022	Turkey	2009-2019	49	79.6	57* (36-82)	HNSCC	All AJCC7	RT, CRT	18.7*	34.7	OS, DFS	L	L	M	L	L	L
Fives	2016	Ireland	2000-2013	54	77.8	NR	OSCC	All NR	RT	45.4* (1-183)	29.6	OS	M	L	L	M	M	L
Ghanem	2017	USA	2010-2015	103	74.8	36-84	HNSCC	All NR	RT, CRT	37* (3-72)	46.6	OS	L	L	M	M	L	L
Ghanem	2019	NR	2005-2015	187	66	58* (24-87)	OSCC	All AJCC7	CRT	NR	61.5	OS, DFS, DMFS	M	L	M	L	L	L
Goyal	2023	India	2010-2020	115	NR	50.11 (11.65)	OSCC	All AJCC8	RT	73*	18	DFS	M	L	M	M	M	L
Holcomb	2023	USA	2000-2019	557	52.8	61.03 (13.9)	OSCC	pT1-2, pN0-1 AJCC7	RT	48* (26-79)	16.7	DFS, LRR	L	L	L	L	L	L
Huang	2019	Taiwan	2006-2013	302	92.7	28-92	OSCC	All AJCC7	RT, CT	NR	48.1	DSS	L	L	M	M	L	L
Huang	2020	Taiwan	2005-2015	258	86	52.7 (11.8)	OSCC	All AJCC7	RT, CT	54 (38.4)	41.9	OS	L	L	L	M	M	L
Huang	2022	China	2013-2017	151	68.2	53* (19-77)	OSCC	T1-2N0 AJCC7	NR	44.2* (3.2-83.0)	41.7	OS, DFS	M	L	M	L	L	L
Huang	2023	China	2013-2020	127	53.5	57* (28-87)	OSCC	All AJCC8	RT	41 (3-111)	18.9	OS	L	L	L	L	L	L
Jang	2016	Korea	2003-2012	295 (OSCC: 166, OPSCC: 71, HPSCC: 41)	75.3	57*	HNSCC	All NR	RT, CRT	32.5 (0-154)	18	DFS	M	L	M	M	M	L
Jardim	2015	Brazil	1998-	142	76.8	57	OSCC	III, IV	NR	31.2*	54.2	OS,	L	L	L	M	L	L

			2009			(22-82)		UICC6		(2-176)		DFS						
Kao	2018	Taiwan	2005-2014	613	90.7	53 (11.38)	OSCC	All AJCC7	RT, CT	NR	33.6	OS	L	L	M	M	L	L
Kim	2021	Korea	2002-2018	275	63.6	58* (18-86)	OSCC	All AJCC8	RT, CRT	40* (5-203)	35.3	OS	L	L	M	M	L	L
Kim	2022	Korea	2001-2020	205	56.6	57* (19-92)	OSCC	All AJCC7	RT, CT	30* (0-234)	18.5	DFS, OS	M	L	M	M	M	L
Lee	2017	Taiwan	2006-2013	396	92.7	53 (11)	OSCC	All AJCC7	RT, CT	28.8 (12-108)	26.3	DFS, DSS	L	L	M	L	L	L
Lee	2020	Korea	2005-2018	291	62.9	63* (24-91)	OSCC	All AJCC8	RT, CT	41 (3-144)	11.3	OS, DFS	L	L	M	L	M	L
Liu	2017	Taiwan	2004-2014	1383	93.8	52.9 (11.1)	OSCC	All AJCC7	RT, CT	42.8 (28.3)	22.7	DSS	L	L	M	M	L	L
Liu	2021	Australia	2008-2013	432	62.7	61* (25-98)	OSCC	All AJCC7	RT	40.8* (6-102)	25.7	DFS, DSS, OS	L	L	M	L	L	L
Lo	2017	Taiwan	2001-2008	105	97.1	55.5 (11.5)	HPSCC	T3-4 AJCC7	NR	50 (34.7)	41.9	DFS, OS, DSS	M	L	M	L	M	L
Maihoefer	2018	Germany	2008-2015	302	74.8	60.7 (20-87)	HNSCC	All UICC7	RT, CT	45*	16.5	OS, DFS, DSS, LR, LRR, DM	L	L	M	L	M	L
Martinez-Flores	2023	Chile	NR	57	50.9	62.8 (13.8)	OSCC	All AJCC8	RT	28* (4-200)	49.1	OS, DSS	M	L	L	L	L	L
Mattavelli	2019	Italy	2000-2014	182	63.2	64* (26-93)	OSCC	All AJCC7	RT, CT	54 (2-163)	49.5	LRFS, DSS, OS, DMFS	L	L	M	L	L	L
Mione	2023	France	2011-2017	167	73	60* (20-94)	OSCC, OPSCC	All UICC7	RT, CT	33*	42	LR, LRR, DMFS	L	L	M	L	L	L
Monterio	2014	Portugal	2000-2010	128	64.8	61.23 (15.57)	OSCC	All NR	RT, CT	33.2 (28.8)	10.9	OS	M	L	M	L	L	L
Na'ara	2023	Israel, India, Italy	1994-2018	1049	61.7	58.4 (14.1)	OSCC	All AJCC8	RT, CT	35.9 (0.1-186.7)	NR	OS, DSS, DFS	M	L	M	L	L	H
Nair	2018	India	2012-	1524	80.4	50*	OSCC	All	NR	22	20.3	OS,	M	L	M	L	L	L

			2015			(19-86)		NR				DFS						
Nguyen	2021	New Zealand	2008-2018	70	57.1	65* (30-91)	OSCC	T1N0 AJCC8	N	55*	14.3	LRR, OS, DFS	L	L	M	M	L	L
Niu	2017	China	2000-2009	207	58.5	64* (15-86)	OSCC	All AJCC7	RT	63* (1-171)	10.6	OS	L	L	L	L	L	L
Otsuru	2023	Japan	2008-2018	564	55.3	60.9 (15.6)	OSCC	I-II AJCC8	CT	59* (1-152)	9.8	LR, DSS, OS	M	L	L	L	L	L
Pant	2021	India	2008-2013	144	85.4	45* (18-85)	OSCC	All AJCC8	RT, CT	87* (60-124)	29	OS, DFS	M	L	M	L	L	L
Park	2020	Australia	2005-2015	116	74.1	50 (34-87)	OSCC	All AJCC7	RT, CRT	NR	47.4	DFS, DSS	L	L	L	M	L	M
Pedersen	2016	Denmark	2007-2013	253	57	63* (30-95)	OSCC	T1-2N0 NR	RT	32* (1-92)	25.3	DFS, DSS	L	L	M	L	M	L
Rodrigues	2020	Brazil	1999-2006	380	76.8	57* (20-93)	OSCC	All AJCC7	RT	51.6* (2.4-120)	22.9	OS, DSS, DFS	L	L	M	M	L	L
Sekikawa	2020	Japan	2007-2016	402	44	67 (23-95)	OSCC	All UICC7	RT, CT	40.3* (2.6-133.0)	10.2	DM	L	L	M	L	L	L
Shibata	2023	Japan	2014-2019	55	56.4	60.7 (15.6)	OSCC	T1-2N0 UICC8	N	37.2 (18.4)	41.8	OS	L	L	M	M	L	L
Shin	2023	Korea	2000-2021	240	90.1	64.8 (42-89)	LSCC	All AJCC8	RT, CRT	65.7* (18-244)	12.5	OS, DFS, LR, DM	L	L	M	L	L	L
Singh	2022	India, Singapore	2006-2013	742	72	53.5 (13.4)	OSCC	III, IV AJCC8	RT, CT	28* (1-177)	56.6	OS	L	L	M	M	L	L
Sinha	2014	USA	1995-2010	95	58.9	61* (22-88)	OSCC	All AJCC7	RT, CT	49* (24-204)	25	DSS, DFS, OS, LR	L	L	M	L	M	L
Spoerl	2022	Germany	2010-2017	493	70.1	NR	OSCC	All UICC7	RT, CT	60*	9.7	OS, DFS	M	L	M	L	L	L
Sridharan	2019	USA, Singapore	1986-2016	494	55	59* (23-88)	OSCC	T1-3 AJCC8	RT, CT	45* (1-348)	32	LR, LRR	L	L	L	L	M	L
Stoop	2020	Nether-land	2000-2017	210	60.5	NR	OSCC	NR	NR	>12	47.1	LRR, DSS	M	L	M	M	L	M
Subramaniam	2019	India	2006-	296	78	55.2*	OSCC	I-II	NR	28*	25.7	LRR	L	L	M	L	L	L

			2014	(I: 154, II: 142)		(31-88)		AJCC8		(6-132)								
Subramaniam	2020	India	2004-2015	425	71.8	45* (18-86)	OSCC	All AJCC7	RT, CRT	27*	31.5	DFS	M	L	M	L	L	L
Tan#	2023	Turkey	NR	73	50.7	65* (28-85)	OSCC	All AJCC8	NR	44.6* (1-155)	24.7	OS	M	L	M	M	L	L
Tan	2023	Turkey	2010-2020	140	93.6	60.9 (9.2)	LSCC	All NR	RT, CT	28* (1-126)	11.4	DFS	M	L	M	M	L	L
Thiagarajan	2014	India	2007-2010	586	71	48*	OSCC	All AJCC6	RT, CT	18*	25.9	DFS	L	L	M	L	L	M
Ting	2021	Taiwan	2002-2010	98	94.9	54 (32-91)	OSCC	T3-4 AJCC7	RT, CT	54.9 (8-166)	44.9	DM, DSS	L	L	L	L	M	L
Trevisani	2023	Brazil	2009-2018	600	73.5	61.3 (11.7)	OSCC	All AJCC8	RT, CT	33.1 (27)	50.3	OS	L	L	M	L	L	L
Xu	2018	China	1999-2011	2036	56.5	59.0 (12.2)	OSCC	All UICC6	RT, CT	65 (1-178)	17.3	DFS, DSS	L	L	M	L	M	L
Xu	2022	USA	1985-2019	232	79.7	58* (34-88)	OPSCC	All AJCC8	RT, CT	45* (3-291)	11	OS, DSS, DFS	L	L	M	M	L	L
Yamada	2021	Japan	2008-2018	215	60.6	64.9 (12.8)	OSCC	All UICC7	RT, CT	56.1 (39.1)	16	OS, DSS	L	L	M	L	L	L
Yang	2018	China	2008-2014	221	55.2	24-75	OSCC	T1-2N0 AJCC6	N	44.3 (21-91)	15.4	LR, DFS, DSS	L	L	L	L	L	L
Zanoni	2019	USA	1985-2015	2082	56	62* (16-100)	OSCC	All AJCC7	RT, CRT	37.6* (1-382)	51	OS, DSS	L	L	M	M	L	L
Zanoni	2022	USA	1998-2015	1369	56	62* (18-100)	OSCC	All AJCC8	RT, CT	39* (1-221)	63	DM	L	L	M	L	M	L
Zhu	2021	China	2008-2017	1272	95.5	NR	LSCC	All AJCC8	RT, CT	64* (36-156)	9.28	OS, DSS, DFS	L	L	M	L	L	L

Key: R: retrospective. P: prospective. NR: not reported. OSCC: oral squamous cell carcinoma. HNSCC: head and neck squamous cell carcinoma. OPSCC: oropharyngeal squamous cell carcinoma. HPSCC: hypopharyngeal squamous cell carcinoma. LSCC: laryngeal squamous cell carcinoma. DSS: disease-specific survival. RT: radiotherapy. CT: chemotherapy. CRT: chemo-radiotherapy. N: no adjuvant treatment. OS: overall survival. DFS: disease-free survival. LR: local recurrence. LRR: locoregional recurrence. LRFS: locoregional relapse free survival. DM: distance metastasis. DMFS: distance metastasis free survival. A: study participation, B: study attrition, C: prognostic factor measurement, D: outcome measurement, E: study confounding, F: statistical analysis and reporting. L: low risk. M: moderate risk. H: high risk. * indicates median value. # distinguishes the study with same first author name and publication year.
